# Cranial acetabular retroversion is common in developmental dysplasia of the hip as assessed by the weight bearing position

**DOI:** 10.3109/17453674.2010.501745

**Published:** 2010-07-16

**Authors:** Anders Troelsen, Lone Rømer, Steffen Jacobsen, Steen Ladelund, Kjeld Søballe

**Affiliations:** ^1^Orthopaedic Research Unit; ^2^Department of Radiology, University Hospital of Aarhus, Aarhus; ^3^Department of Orthopedics, University Hospital of Hvidovre, Copenhagen; ^4^Clinical Research Centre, University Hospital of Hvidovre, CopenhagenDenmark

## Abstract

**Background and purpose:**

The appearance of acetabular version differs between the supine and weight bearing positions in developmental dysplasia of the hip. Weight bearing radiographic evaluation has been recommended to ensure the best coherence between symptoms, functional appearance, and hip deformities. Previous prevalence estimates of acetabular retroversion in dysplastic hips have been established in radiographs recorded with the patient supine and with inclusion only if pelvic tilt met standardized criteria. We assessed the prevalence and the extent of acetabular retroversion in dysplastic hip joints in weight bearing pelvic radiographs.

**Patients and methods:**

We assessed 95 dysplastic hip joints (54 patients) in weight bearing anteroposterior pelvic radiographs, measuring the acetabular height and the distance from the acetabular roof to the point of crossing of the acetabular rims, if present.

**Results:**

Acetabular retroversion was found in 31 of 95 dysplastic hip joints. In 28 of 31 hip joints with retroversion, crossover of the acetabular rims was positioned within the cranial 30% sector. The degree of pelvic tilt differed between retroverted and non-retroverted dysplastic hip joints, though only reaching a statistically significant level in male dysplastic hip joints.

**Interpretation:**

We identified cranial acetabular retroversion in one-third of dysplastic hip joints when assessed on weight bearing pelvic radiographs. If assessed on pelvic radiographs obtained with the patient supine, and with inclusion only if the degree of pelvic tilt meets standardized criteria, the prevalence of acetabular retroversion may be underestimated.

## Introduction

It is important to recognize acetabular deformities associated with developmental dysplasia of the hip (DDH), especially when planning a joint preserving periacetabular osteotomy (PAO). It is commonly perceived that DDH is associated with lateral and anterior acetabular deficiency, with the acetabulum oriented in abnormal anteversion (Anda et al. 1991a,b, Nakamura et al. 2000, Jacobsen et al. 2005, 2006, Jacobsen 2006).

It has been found recently that acetabular retroversion co-exists in some hip joints with DDH, causing excessive anterior coverage (Li and Ganz 2003, Mast et al. 2004, Ezoe et al. 2006). Furthermore, it has been shown that the radiographic appearance of the acetabular version depends on the degree of pelvic tilt and that the appearance of acetabular version differs between the supine and weight bearing positions (Siebenrock et al. 2003, Troelsen et al. 2008). It is debated whether the hip joint deformities of DDH, including acetabular version, should be assessed in supine or weight bearing pelvic radiographs. It is most usual to use radiographs recorded with the patient supine, but recently it has been recommended that weight bearing pelvic radiographs should be recorded to ensure the best agreement between symptoms, functional appearance, and hip deformities in DDH (Troelsen et al. 2008). Assessment of acetabular version is an important step when planning a PAO in which the acetabulum is re-oriented, and the preoperative planning of the amount of redirection necessary may be affected depending on the position of the patient during the radiographic examination.

As previous assessments of the acetabular version in DDH hip joints have been made in supine pelvic radiographs with inclusion only if the degree of pelvic tilt met standardized criteria (Li and Ganz 2003, Mast et al. 2004, Ezoe et al. 2006), and given the different appearances of acetabular version in the supine and weight bearing positions (Troelsen et al. 2008), we found it necessary to evaluate acetabular retroversion in dysplastic hip joints in weight bearing pelvic radiographs. We defined 4 research aims: (1) assessment of the prevalence of acetabular retroversion in DDH hip joints in weight bearing pelvic radiographs, (2) quantification of the extent of acetabular retroversion; if present, investigation of any relationships between the degree of (3) hip dysplasia or (4) pelvic tilt and the presence or extent of retroversion.

## Patients and methods

All preoperative weight bearing anteroposterior pelvic radiographs taken from March 2006 through December 2008 in patients scheduled for PAO were eligible for inclusion and were retrospectively reviewed (n = 93 pelvic radiographs/patients). Patients were excluded if previous surgery had been performed on either hip (n = 15 PAOs; n = 3 total hip replacements), if dysplasia was associated with subluxation with a broken Shenton line in either hip (n = 8), if dysplasia was associated with femoral head deformity (n = 7 Legg-Calvé-Perthes disease), if either joint showed signs of osteoarthritis corresponding to Tönnis grades 2 or 3 (n = 1) (Tönnis 1987), if the acetabular rims could not be identified in any of the hip joints (n = 1), or if the acetabulum was excessively rotated corresponding to a foramen obturator index of Tönnis outside 0.7–1.8 (Jacobsen et al. 2004) (n = 0). Thus, the study group was restricted to 58 pelvic radiographs/patients. Further exclusion of hip joints in which the acetabular rims could not be identified (n = 7) or in which the center-edge angle of Wiberg (1939) was ≥ 25° (n = 14) defined the final study group of 95 hip joints in 54 pelvises/patients (13 unilateral DDH and 41 bilateral DDH). There were 10 males and 44 females with a mean age of 36 (14–57) years. In the hip joints, the median center-edge angle of Wiberg was 16° (0–24) and the median acetabular index angle of Tönnis was 13° (2–27). The mean foramen obturator index of Tönnis was 1.07 (0.83–1.53).

Weight bearing anteroposterior pelvic radiographs were recorded according to a previously described protocol (Troelsen et al. 2008): The patients were asked to stand with the lower extremities parallel and with enough internal rotation for the feet to touch each other, thus obtaining 15–20° of internal rotation. They were instructed to bear equal weight on both extremities. We presumed that the natural or functional appearance of the pelvic position with respect to tilt is maintained. The X-ray beam was directed to the midpoint between the symphysis and the center between both anterior superior iliac spines.

**Figure 1.**
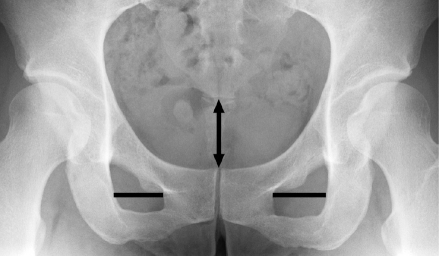
Section of a weight bearing anteroposterior pelvic radiograph showing the measurement of pelvic tilt and rotation. Pelvic tilt is assessed by measuring the distance from the upper edge of the symphysis to the sacrococcygeal joint (vertical line with arrowheads). Pelvic rotation is assessed by measuring the widths of the obturator foramens (the horizontal lines) and calculating the foramen obturator index (right foramen width divided by left foramen width).

**Figure 2.**
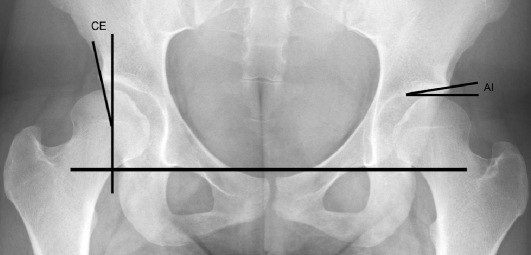
Section of a weight bearing anteroposterior pelvic radiograph showing the assessment of the center-edge angle of Wiberg (Wiberg 1939) (marked “CE”) and the acetabular index angle of Tönnis (Tönnis 1987) (marked “AI”). The teardrop line (the horizontal line interconnecting the hip joints) is the line of reference preceding construction of the angles.

**Figure 3.**
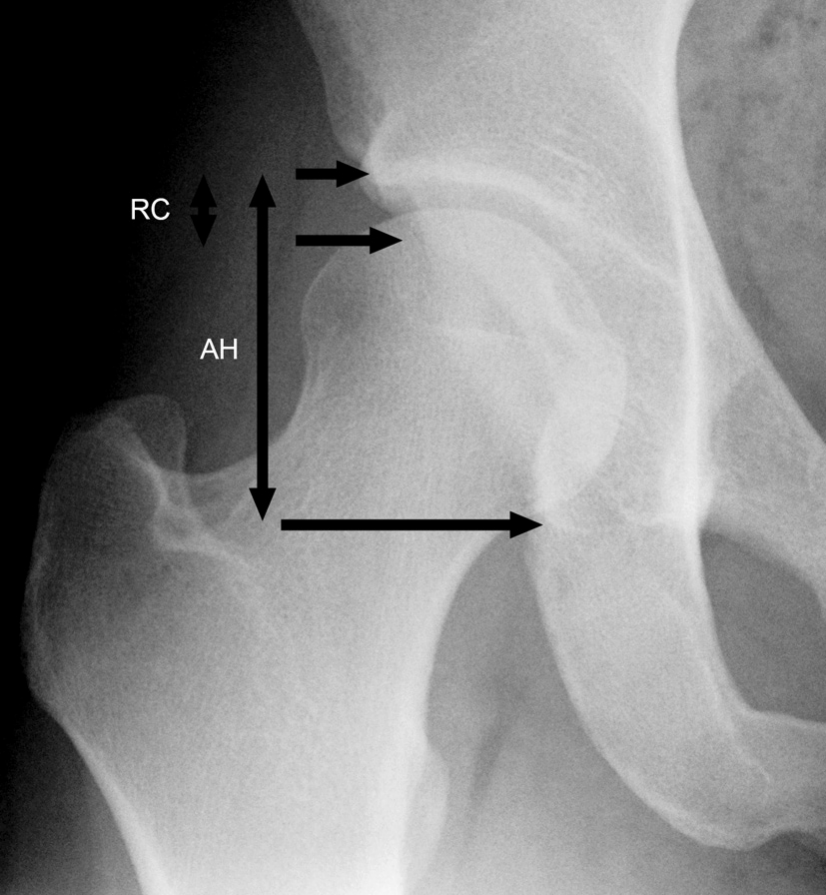
Section of a weight bearing anteroposterior pelvic radiograph showing the right hip. The most cranial arrow marks the acetabular roof, the arrow in the middle marks the crossover of acetabular rims, and the caudal arrow marks the termination of the posterior rim. The acetabular height is marked “AH”, and the distance from the acetabular roof to the crossover of acetabular rims is marked “RC”. To take into consideration differences in acetabular heights, the extent of retroversion (the distance RC) was expressed as a percentage of the acetabular height (AH).

Assessment of pelvic tilt and rotation was made in each pelvic radiograph, and in each hip joint the center-edge angle of Wiberg (1939), the acetabular index angle of Tönnis (1987), and acetabular version were assessed (using the digital distance-measuring and angle-measuring tools of the DS 3000 Workstation; Agfa Healthcare, Holte, Denmark). Assessments were performed by one observer (AT). Pelvic tilt was measured as the distance from the upper edge of the symphysis to the sacrococcygeal joint (Siebenrock et al. 2003) (Figure 1). Inclination is defined as a forward rotation or flexion of the pelvis around a transverse axis through the hips (this will increase the distance from the symphysis to the sacrococcygeal joint) and reclination or extension as a backward rotation around the same axis (this will decrease the distance from the symphysis to the sacrococcygeal joint) (Anda et al. 1990). Pelvic rotation was assessed by measuring the widths of the obturator foramens and then calculating the foramen obturator index of Tönnis (1987) (Figure 1). The center-edge angle of Wiberg and the acetabular index angle of Tönnis were measured as previously described (Figure 2). We measured the acetabular version by assessment of the acetabular rims and, if present, the crossover sign was identified (Reynolds et al. 1999). The distances from the acetabular roof to the caudal termination of the posterior rim (the acetabular height) and from the acetabular roof to the point of crossover of the acetabular rims (extent of retroversion) were measured. To take into consideration differences in acetabular heights, the extent of retroversion was calculated and expressed as a percentage of the acetabular height (Figure 3). Intra- and interobserver variability assessment of the acetabular height, the extent of retroversion, and the distance from the upper edge of the symphysis to the sacrococcygeal joint (Siebenrock et al. 2003) was performed in a randomly selected subset of 30 hips (30 patients). Measurements for interobserver variability assessment were performed by a senior consultant radiologist (LR).

### Statistics

Continuously distributed data are presented as mean with range and compared using gee methods (Liang and Zeger 1986) to compensate for correlation between both hips from the same patient in calculations of p-values. Difference in proportions was assessed using gee-logistic regression. Intra- and interobserver assessments were analyzed by a Bland-Altman approach (Bland and Altman 2003) and presented as difference of the mean (diff. mean), standard deviation (SD), and 95% limits of agreement (95% LOA). According to this approach, 95% of differences between two series of measurements can be expected to lie within the “95% limits of agreement”. Statistical analysis was performed using the Stata software package version 10.1 (StataCorp, College Station, TX) and the statistical environment R-2 10.0, including the geepack package.

## Results

The intra- and interobserver variability of the acetabular height (cm) was: diff. mean 0.06, SD 0.20, 95% LOA -0.33–0.46 and diff. mean -0.15, SD 0.16, 95% LOA -0.48–0.17, respectively. The intra- and interobserver variability of the extent of retroversion (in cm) was: diff. mean 0.04, SD 0.15, 95% LOA -0.27–0.34 and diff. mean -0.06, SD 0.11, 95% LOA -0.27–0.16, respectively. Assessing the presence of a crossover sign showed 100% agreement between observations on intra- and interobserver variability assessment. The intra- and interobserver variability of the extent of retroversion as a percentage of the acetabular height was: diff. mean 0.01, SD 0.03, 95% LOA -0.05–0.06 and diff. mean -0.01, SD 0.02, 95% LOA -0.05–0.04, respectively. The intra- and interobserver variability of the distance from the upper edge of the symphysis to the sacrococcygeal joint (cm) was: diff. mean 0.02, SD 0.10, 95% LOA -0.18–0.22 and diff. mean -0.05, SD 0.31, 95% LOA -0.65–0.56, respectively.

Acetabular retroversion, identified by the crossover sign, was found in 31 of 95 dysplastic hip joints (33%). In unilateral dysplastic hip joints (n = 13) acetabular retroversion was found in 7 hips, and in bilateral dysplastic hip joints (n = 82) acetabular retroversion was found in 24 hips. The prevalence of acetabular retroversion was not statistically significantly different between hip joints of males (5 of 18 hips) and hip joints of females (26 of 77 hips) (p = 0.7).

In 28 of 31 dysplastic hip joints with retroversion, the crossover of the acetabular rims was positioned within the cranial 30% of the acetabular height. Crossover of the acetabular rims never occurred more caudal than the cranial 41% of the acetabular height (Table). In unilateral dysplastic hip joints, the crossovers of acetabular rims were positioned in the cranial 8–27% of the acetabular height and in bilateral dysplastic hip joints the crossovers of acetabular rims were positioned in the cranial 5–41% of the acetabular height.

**Table:** The distribution of the extent of acetabular retroversion in dysplastic hip joints

Distance from the acetabular roof to the point of acetabular rim crossover, in % of acetabular height	Number of dysplastic hip joints (total n = 95)
No retroversion	64
0–10	9
11–20	12
21–30	7
31–40	2
41–50	1
51–100	0

Comparison of the center-edge angle in dysplastic hip joints with (median: 18°; range: 6–24°) and without (median: 16°; range: 0–24°) retroversion did not reveal any statistically significant difference between the groups (p = 0.2). In dysplastic hip joints with retroversion, linear regression analysis did not show any statistically significant relationship between the degree of hip dysplasia (expressed as the center-edge angle) and the extent of retroversion (p = 0.44; R² = 0.02).

The distance from the upper edge of the symphysis to the sacrococcygeal joint differed statistically significantly between males (mean: 2.8 cm; range: 0.7–4.7 cm) and females (mean: 4.9 cm; range: 1.5–8.0 cm) (p < 0.001). Comparison of the degree of pelvic tilt in female dysplastic hip joints with (mean: 5.4 cm; range: 2.6–8.0 cm) and without (mean: 4.6 cm; range: 1.5–6.8 cm) retroversion did not show any statistically significant difference between the groups (p = 0.2). Comparison of the degree of pelvic tilt in male dysplastic hip joints with (mean: 4.1 cm; range: 3.6–4.7 cm) and without (mean: 2.4 cm; range: 0.7–4.1 cm) retroversion showed that there was a statistically significant difference between the groups (p = 0.002).

**Figure 4.**
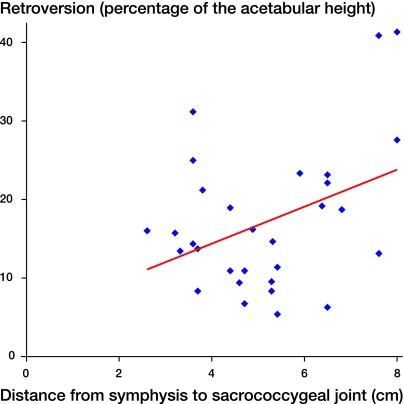
A scatter plot with the distance from the symphysis to the sacrococcygeal joint plotted on the x-axis, and the extent of retroversion, expressed as a percentage of the acetabular height, plotted on the y-axis. The regression line is shown in red (p = 0.004; R^2^ = 0.15).

In dysplastic hip joints with retroversion, linear regression analysis showed that there was a statistically significant relationship between the degree of pelvic tilt and the extent of retroversion (p = 0.004; R² = 0.15) (Figure 4); i.e. increased pelvic inclinations were related to greater extents of acetabular retroversion. Performance of the same linear regression analysis in female dysplastic hip joints alone did not show a similar statistically significant relationship (p = 0.12; R² = 0.20).

## Discussion

Previous assessments of acetabular retroversion in dysplastic hip joints have been made on supine radiographs with inclusion only if the degree of pelvic tilt met standardized criteria (Li and Ganz 2003, Siebenrock et al. 2003, Mast et al. 2004, Ezoe et al. 2006). However, pelvic tilt and the assessment of acetabular retroversion have been shown to differ between the supine and weight bearing positions (Troelsen et al. 2008). It has therefore been recommended that weight bearing pelvic radiographs should be obtained for the best coherence between symptoms, functional appearance, and hip deformities in DDH (Troelsen et al. 2008). We therefore found it necessary to evaluate the prevalence and the extent of acetabular retroversion in dysplastic hip joints on weight bearing pelvic radiographs. Furthermore, we assessed whether there were any relationships between the degree of hip dysplasia or pelvic tilt and the presence or the extent of acetabular retroversion in dysplastic hip joints.

Full-scale assessment of the bony structures in the pelvis is achieved by performing a 3-D CT scan. Thus, the assessment of pelvic tilt and acetabular version using 2-D plain radiographs that we performed in the present study might not be accurate. However, a moderately strong correlation between the distance from the upper edge of the symphysis to the sacrococcygeal joint on anteroposterior pelvic radiographs and the pelvic tilt has been shown (Tannast et al. 2006), and the crossover sign has been shown to be highly valid in the assessment of acetabular retroversion on anteroposterior pelvic radiographs (Jamali et al. 2007). Furthermore, CT scans are made with the patient supine, and therefore—as with plain radiographs obtained with the patient supine—it does not reflect the functional position of the pelvis. It seems appropriate to assess pelvic tilt and acetabular retroversion on weight bearing pelvic radiographs. For full-scale 3-dimensional preoperative assessment of the complex patho-anatomy, a CT scan is preferred by many surgeons.

Even though the distance from the upper edge of the symphysis to the sacrococcygeal joint on anteroposterior pelvic radiographs has been shown to have a moderately strong correlation with the pelvic tilt (Tannast et al. 2006), the assessment of pelvic tilt could be further improved by obtaining a lateral view of the pelvis. However, a weight bearing lateral recording of the pelvis is often not performed in daily clinical practice as it is purely diagnostic of the degree of pelvic tilt. A specially constructed inclinometer, applied directly to the patient on the anterior superior iliac spines and the pubic tubercles, has previously been reported to be reliable in assessing the degree of pelvic tilt (Anda et al. 1990). Such an inclinometer may be used in clinical studies. Pelvic rotation is known to influence the measurement of interconnecting lines in the pelvis (Foss et al. 2007), and it is likely that excessive rotation may influence the appearance of acetabular version in anteroposterior pelvic radiographs. In the present study, we applied limits of pelvic rotation to exclude excessively rotated pelvises, thus minimizing bias in the assessment of acetabular version. Finally, the intra- and interobserver variability assessment showed good agreement between observations with narrow 95% limits of agreement.

Li and Ganz (2003) and Ezoe et al. (2006) reported prevalences of acetabular retroversion of 17% and of 18%, respectively. Li and Ganz (2003) included only pelvic radiographs if the distance from the coccyx to the symphysis was between 0 and 2 cm, and Ezoe et al. (2006) recorded their radiographs with the patients supine and pelvic radiographs were only included if the measurements of pelvic tilt met previously suggested standardized criteria (Siebenrock et al. 2003). With increasing degrees of pelvic inclination, the appearance of acetabular retroversion gets more pronounced (Siebenrock et al. 2003). Thus, if pelvises with higher degrees of inclination (long distances from the upper edge of the symphysis to the sacrococcygeal joint or to the tip of the coccyx) are excluded from evaluation, this will lead to exclusion—and thereby underestimation—of hips with the appearance of retroversion. We assessed the pelvises in their natural or functional appearance and none were excluded. Thus, it is not surprising that the prevalence of acetabular retroversion in dysplastic hip joints of 33% in the present study is higher than previous estimates. Mast et al. (2004) found a prevalence of acetabular retroversion in dysplastic hip joints of 37%. This finding is similar to ours, which is surprising as only pelvises/hip joints with a distance from the tip of the coccyx to the symphysis of ≤ 2 cm were included. One possible explanation might be differences in magnification of radiographs between studies. With little magnification of the radiographs, it is possible that pelvises with extensive inclination would also be included even if the discriminator is a distance of ≤ 2 cm from the tip of the coccyx to the symphysis.

It has been reported that the diagnosis of retroversion in DDH depends on the relationship of the anterior and posterior walls in the cranial one-third of the acetabulum (Li and Ganz 2003). We found a similar pattern, as 28 of 31 dysplastic hip joints with retroversion showed crossover of the acetabular rims within the cranial 30% of the acetabular height. As the pattern of a cranially localized acetabular retroversion appears to be constant in dysplastic hip joints, it seems likely to be a developmental pathomorphological feature that is expressed in some dysplastic hip joints. A great contributor to the development of cranial acetabular retroversion may be altered tensile forces across the hip joint, which may result in a local prominence of the anterior acetabular rim, and not retroversion of the entire acetabular socket. Previous studies using 3-D CT scan have mainly described hip dysplasia as a condition with anterolateral deficiency and acetabular anteversion (Anda et al. 1991a,b, Nakamura et al. 2000, Jacobsen et al. 2005, 2006, Jacobsen 2006). However, the measurements of acetabular version were done at a level through the center of the femoral head, and therefore cranial retroversion was not detected.

We found that dysplastic hips with retroversion were associated with statistically significantly increased pelvic inclination compared to dysplastic hips with no signs of retroversion. Furthermore, we found that there was a correlation between the extent of acetabular retroversion and the degree of pelvic tilt. This is in accordance with previous findings in a methodological study on cadaver pelvises (Siebenrock et al. 2003). The finding is easily explained, as increased pelvic inclination will result in increased projection of the prominent anterior rim in the caudal direction (i.e. increased extent of acetabular retroversion). However, the R^2^ value of the correlation was only 0.15, meaning that only a minor part of the correlation can be explained by variance in the degree of pelvic tilt. This may be accounted for in part by anatomical heterogeneity in the pelvises and hip joints that were assessed. Considering female dysplastic hips alone, the correlation did not reach statistically significant levels. In our study, the center-edge angle was not statistically significantly different between dysplastic hip joints with and without retroversion. This is in accordance with the findings of a previous study on dysplastic hip joints that compared the center-edge angle in acetabuli with anteversion and retroversion (Mast et al. 2004).

In general, acetabular retroversion is thought to be a cause of hip pain and a precursor of osteoarthritis (Giori and Trousdale 2003, Kim et al 2006, Ganz et al. 2008). It seems obvious that cranial retroversion in dysplastic hip joints could be the cause of anterolateral impingement and tearing of the labrum. However, the importance of cranial acetabular retroversion in dysplastic hip joints remains uninvestigated. Recognition of acetabular retroversion is important during preoperative planning and during intraoperative evaluation of the acetabular reorientation achieved when performing a periacetabular osteotomy. If not recognized, there is a risk that attempts to correct the anterolateral deficiency of a dysplastic hip by means of a periacetabular osteotomy will result in femoro-acetabular impingement and continued risk of development of osteoarthritis of the hip joint.

We believe that good agreement between symptoms, functional appearance, and hip deformity in dysplastic hip joints is assured by obtaining a weight bearing pelvic radiograph. We identified cranial acetabular retroversion in one-third of dysplastic hip joints when assessed on weight bearing pelvic radiographs. If assessed on pelvic radiographs obtained with the patient in supine position and with inclusion only if the degree of pelvic tilt meets standardized criteria, the prevalence of acetabular retroversion may be underestimated.
